# Energy and Health Efficiencies in China with the Inclusion of Technological Innovation

**DOI:** 10.3390/ijerph16214225

**Published:** 2019-10-31

**Authors:** Qian Wang, Duo Li, Tzu-Han Chang

**Affiliations:** 1 Public Sector Research Center KRI, School of Economics, Jilin University, Qianjin Street 2699, Changchun 130012, China; liduo@jlu.edu.cn; 2 Department of Economics, Soochow University, No. 56, Kueiyang St., Sec. 1, Taipei 100, Taiwan; angleyc06@gmail.com

**Keywords:** SMB VRS two-stage DEA, energy efficiency, health efficiency, technological innovation

## Abstract

The price people pay for low energy efficiency includes not only high manufacturing costs, but also public health. With technological innovation as the driving factor for improving energy efficiency, this study uses two-stage dynamic undesirable data envelopment analysis (TDU-DEA) under variable return to scale to evaluate energy and health efficiencies with inclusion of technological innovation in 30 provinces of China over the period 2013–2016. The results show that the mean overall efficiencies and ranks in the eastern region are significantly higher than those in the non-eastern region, with or without the inclusion of technological innovations, and that energy efficiency in most provinces is higher than health efficiency. The average technological innovation efficiencies for energy conservation are higher than those for respiratory medical treatment. The former gap between the eastern region and non-east region is also smaller than the latter. Lastly, regions with the best technological innovation efficiencies are Beijing, Shanghai, Guangdong, Fujian, Hainan, Hebei, Inner Mongolia, Ningxia, Qinghai, Shandong, Shanxi, Tianjin, Xinjiang, and Yunnan.

## 1. Introduction

Both higher manufacturing cost and more health treatment expenditure are conditions that people pay for lower energy efficiency. With a huge consumption of fossil fuels, the economic growth rate of China has been above 9% for nearly forty years. At the same time, the lung cancer mortality rate in China has increased from 7.09 per 100,000 persons in 1975 to 30.83 in the latest census in 2005, although mortality rates of gastric cancer and liver cancer have not risen as much. Among all diseases leading to neonatal death, that of pneumonia is at the top [1]. Undoubtedly, air pollution caused by the combustion of fossil fuels like coal and oil account for some or most of these phenomena.

In 2013, the Helsinki statement on health in all policies proposed a new cross-sectional public policy framework. In that same year the State Council of China issued its 12^th^ five-year plan for energy development, implementing many energy policies such as the price reforms of natural gas, electricity, etc. We thus analyze jointly provincial energy and health efficiencies with the inclusion of technological innovation in China during 2013–2016 to provide evidence on the associations between energy, air pollution, health, and technological innovation.

As renewable energy cannot replace traditional fossil fuels in a short time, energy efficiency has attracted a lot of scholars’ attention. Some researchers focus on measuring energy efficiency [2,3,4,5,6,7,8,9,10], while others target the influence factors of energy efficiency [11,12,13,14,15]. In the latter field, most researchers emphasize the effect of technological innovation on energy efficiency [16,17,18]. In fact, a positive influence of technological innovation on energy efficiency is found within different industries [19,20,21].

The effects of pollutants on human health have widely appeared in the literature, especially the relationship between air pollutants, such as carbon dioxide, sulfur dioxide, dust, nitrogen oxides, PM10 and PM2.5, and respiratory disease [22,23,24,25,26,27,28,29,30]. PM 2.5 denotes PM2.5 particles, which are air pollutants with a diameter of 2.5 micrometers or less. PM 10 denotes PM10 particles, which are air pollutants with a diameter of 10 micrometers or less. These particles generally come from activities that burn fossil fuels. Many empirical researchers have looked at the impact of air pollution on human health from various main cities, provinces, and regions in China [31,32,33,34,35]. Some scholars find that low energy efficiency in China is related to high air pollution, which is harmful to public health [36].

Data Envelopment Analysis (DEA) has been adopted widely to analyze energy efficiency and health efficiency. Tone [37], Fare et al. [38], and Tone and Tsutsui [39,40] used a two-stage DEA or Network slacks-Based Measure to explore the efficiency in the production stage and pollution treatment stage.

There are, however, less studies jointly focused on the relationships between energy, environmental pollution, health, and technological innovation. In this paper, we add stock of energy conservation knowledge as input variables like labor and energy consumption in the production stage (stage 1) and stock of respiratory medical treatment knowledge in the health treatment stage (stage 2). The undesirable intermediate outputs (CO_2_, PM2.5, and PM10) in stage 1 are regarded as inputs in stage 2. Mortality rate and respiratory disease rate are regarded as outputs in stage 2. Thus, the relationship between energy, environmental pollution, health, and technological innovation are constructed using a two-stage DEA model.

This paper provides two contributions. It is the first empirical study on energy and health efficiencies with the inclusion of technological innovation. Through International Patent Classification (IPC) codes, we construct patent data on energy conservation and respiratory medical treatment and then construct stock of knowledge by simultaneously considering two issues of decay and diffusion of a patent. Technological innovation is the driving force for technology progress, which is the source of efficiency improvement, and is regarded as input in both the production stage and health treatment stage in a two-stage DEA model. Secondly, we extend the two-stage dynamic undesirable DEA under constant return to scale (CRS) to variable return to scale (VRS). Most environmental pollution and energy efficiency analyses are usually conducted under CRS, ignoring the role of knowledge. According to the New Growth theory (Romer, 1986 [41]), knowledge, unlike land and capital, can create increasing profit. The spillover effects of technological innovations enhance aggregate TFP in the long-run (Chen and Wemy, 2015 [42]). We take knowledge as an input variable. Therefore, the VRS research framework is used to evaluate energy, environmental, and health efficiencies.

The remainder of this paper is organized as follows. Section 2 gives the literature review. Section 3 describes the research method. Section 4 presents the empirical results and discussion. Section 5 offers the conclusions.

## 2. Literature Review

There is an abundant amount of literature on energy environmental problems and health issues. Most studies take them as two separate issues.

Research on energy environment issues has typically focused on energy and environmental efficiencies in recent years. Using the DEA approach, some studies have noted that energy and emission efficiencies in European countries are higher than that in Asian countries (Lu et al. [2]; Wang et al. [3]; Guo et al. [4]). Song et al. [5] found that BRICS (Brazil, Russia, India, China, South Africa) are less energy efficient, but are improving fast. There are many studies on energy efficiency in China. Some scholars showed energy and emission efficiencies in its eastern region are higher than that in the central and western regions by DEA (Hu et al. [6]; Wang et al. [7]; Yao et al. [8]; Meng et al. [9]). Some have presented that the gap has widened rapidly since 2001 (Wang et al. [10]).

The literature has further explored the influencing factors of energy efficiency. Some emphasized that economic development has a positive relationship with energy efficiency (Qin et al. [11]; Wang et al. [12]; Du et al. [13]). Some explored the influence of market-oriented reform, especially the promotion of a factor market, on energy and emission efficiencies (Lin and Du [14]). Some studied energy efficiency from the aspect of a circular economy (Halkos et al. [15]), while others focused on the important role of technical progress and technological innovation on energy efficiency (Feng et al. [16]; Jaffe et al. [17] Wang et al. [18]). Still, others examined the positive influence of technological innovation on energy efficiency in different industries, such as the air conditioning industry (Newell et al. [19]) and automotive industry (Crabb and Johnson [20]; Hascic et al. [21]).

Studies on environmental health issues have focused on the influence of environmental pollution on public health. Scholars found that air pollution (particulate matter) affects asthma and lung functions (Knibbs et al. [22]; Dauchet et al. [23]) and increases the risks of many diseases. These diseases are lung carcinogenesis (Santibanez-Andrade et al. [24]), arterial stiffness (Ljungman et al. [25]), Parkinson’s disease (Kasdagli et al. [26]), asthma (Khreis et al. [27]), hypertension (Kim et al. [28]), and communicable diseases (Landrigan et al. [29]). Air pollution increases the weekly morbidity in California by 0.5–4.6% (Ngo et al. [30]). Some scholars have explored air pollution in provinces and cities of China, finding that it relates to 6.92% of China’s total deaths in 2015 (Maji et al. [31]), respiratory disease (Chen et al. [32]; Chen et al. [33]), hypertension (Yang et al. [34]), and public health (Wang et al. [35]).

Chen et al. [36] studied jointly the energy and health efficiencies in China by a two-stage dynamic undesirable DEA for the first time. Compared to Chen et al. [36], who employed media reports as the input variable in DEA, we provide additional evidence on the energy and health efficiencies in China by including technological innovations. Moreover, different from Chen et al. [36], who used the DEA model under constant returns to scale, we employ the DEA model under variable returns to scale. There is no literature studying energy and health efficiencies with the inclusion of technological innovation. This paper fills this research gap.

## 3. Research Method

Tone [37] proposed a slack model that uses Slack-Based measure (SBM), taking into account the slack between input and output terms and presenting SBM efficiencies in terms of a non-radial variable estimation with an efficiency value between 0 and 1. In 2007, Färe, Grosskopf, and Whittaker proposed the network data envelopment analysis (NDEA), which recognized that the production process is composed of many sub-production technologies (Sub-DMU), and then used a traditional model to find the best solution. Following Färe et al. [38], Tone and Tsutsui [39] proposed a weighted slack-based measures NDEA model in 2009, in which the decision-making unit linkages between the divisions are used as the foundation for analyzing the network DEA model. In 2013, Tone and Tsutsui [40] set up a weighted slack-based measures dynamic network DEA model, in which the decision-making unit linkages are used as the basis for analyzing the network DEA model with each division being seen as a sub-DMU. Since this study considers undesirable outputs in the dynamic network DEA model, we can modify dynamic network DEA to be two-stage dynamic undesirable data envelopment analysis.

Following Chen et al. [36], our study uses two-stage dynamic undesirable data envelopment analysis (TDU-DEA) to evaluate the energy efficiency and health efficiency with the inclusion of technological innovation in 30 provinces of China over the period 2013–2016, in which there are two stages. The first-stage inputs are labor, energy consumption, and stock of energy conservation knowledge, the output is gross domestic product (GDP), and the link variables to the second stage are PM2.5, PM10, and CO_2_ emissions. The second-stage inputs are stock of energy conservation knowledge and stock of respiratory medical treatment knowledge, the outputs are mortality rate and respiratory disease rate, and the carryover variable is fixed assets investment. This paper focuses on the association of technological innovations with energy and health efficiencies. Based on the New Growth Theory (Romer [41]), we choose variable returns-to-scale (VRS) DEA and designed two-stage dynamic undesirable data envelopment analysis (TDU-DEA) under VRS as follows.

Suppose there are *n* DMUs (*j* = 1,…,*n*), with each having *k* divisions (*k* = 1,…,K), and T time periods (*t* = 1,…,T). Each DMU has an input and output at time period t and a carryover (link) to the next *t*+1 time period.

Set $m_{k}$ and $r_{k}$ to represent the inputs and outputs in each division K, with (k,h)i representing divisions *k* to *h* and $L_{hk}$ being the *k* and *h* division set. The inputs, outputs, links, and carryover definitions are outlined in the following paragraphs. The following is the non-oriented model.

(a) Objective Function

Overall Efficiency:

Subject to:

(1) $$x_{ok}^{t}=X_{k}^{t}\lambda_{k}^{t}+s_{ko}^{t-}\left(\forall k,\forall t\right)$$

(2) $$y_{okgood}^{t}=Y_{kgood}^{t}\lambda_{k}^{t}-s_{kogood}^{t+}\left(\forall k,\forall t\right)$$

(3) $$y_{okbad}^{t}=Y_{kbad}^{t}\lambda_{k}^{t}+s_{kobad}^{t-}\left(\forall k,\forall t\right)$$

(4) $$e\lambda_{k}^{t}=1\left(\forall k,\forall t\right)$$

(5) $$\lambda_{k}^{t}\geq 0,s_{ko}^{t-}\geq 0,s_{kogood}^{t+}\geq 0,s_{kobad}^{t-}\geq 0,\left(\forall k,\forall t\right)$$

(6) $$Z_{o(kh)in}^{t}=Z_{(kh)in}^{t}\lambda_{k}^{t}+S_{o(kh)in}^{t}\left((kh)in=1,\ldots ,linkin_{k}\right)$$

(7) $$\sum_{j=1}^{n}z_{jk_{1}\alpha }^{\left(t,(t+1)\right)}\lambda_{jk}^{t}=\sum_{j=1}^{n}z_{jk_{1}\alpha }^{\left(t,(t+1)\right)}\lambda_{jk}^{t+1}\left(\forall k;\forall k_{l};t=1,\ldots ,T-1\right)$$

(8) $$Z_{ok_{l}input}^{\left(t,(t+1)\right)}={\sum_{j=1}^{n}z}_{jk_{l}input}^{\left(t,(t+1)\right)}\lambda_{jk}^{t}+s_{ok_{l}input}^{\left(t,(t+1)\right)}k_{l}=1,\ldots ,ngood_{k};\forall k;\forall t)$$

(9) $$s_{ok_{l}good}^{\left(t,(t+1)\right)}\geq 0,\left(\forall k_{l};\forall t\right)$$

(b) Period and Division Efficiencies

Period and division efficiencies are as follows:

(b1) Period Efficiency:

(10) $$\partial_{0}^{*}=\min\frac{\sum_{k=1}^{K}W^{k}\left[1-\frac{1}{m_{k}+linkin_{k}}(\sum_{i=1}^{m_{k}}\frac{S_{iok}^{t-}}{x_{iok}^{t}}+\sum_{{\left(kh\right)}_{l}=1}^{linkin_{k}}\frac{s_{o{\left(kh\right)}_{l}in}^{t}}{z_{o{\left(kh\right)}_{l}in}^{t}})\right]}{\sum_{k=1}^{K}W^{k}\left[1+\frac{1}{r_{1k}+r_{2k}+ngood_{k}}(\sum_{r=1}^{r_{1k}}\frac{s_{rokgood}^{t+}}{y_{rokgood}^{t}}+\;\sum_{r=1}^{r_{2k}}\frac{s_{rokbad}^{t-}}{y_{rokbad}^{t}}+\sum_{k_{l}}^{ngood_{k}}\frac{s_{ok_{l}good}^{\left(t,t+1\right)}}{z_{ok_{l}good}^{\left(t,t+1\right)}})\right]}$$

(b2) Division Efficiency:

(11) $$\varphi_{0}^{*}=\min\frac{\sum_{t=1}^{T}W^{t}\left[1-\frac{1}{m_{k}+linkin_{k}+ninput_{k}}(\sum_{i=1}^{m_{k}}\frac{S_{iok}^{t-}}{x_{iok}^{t}}+\sum_{{\left(kh\right)}_{l}=1}^{linkin_{k}}\frac{s_{o{\left(kh\right)}_{l}in}^{t}}{z_{o{\left(kh\right)}_{l}in}^{t}}+\sum_{k_{l}}^{ninput_{k}}\frac{s_{ok_{l}input}^{\left(t,t+1\right)}}{z_{ok_{l}input}^{\left(t,t+1\right)}})\right]}{\sum_{t=1}^{T}W^{t}\;\left[1+\frac{1}{r_{1k}+r_{2k}}(\sum_{r=1}^{r_{1k}}\frac{s_{rokgood}^{t+}}{y_{rokgood}^{t}}+\;\sum_{r=1}^{r_{2k}}\frac{s_{rokbad}^{t-}}{y_{rokbad}^{t}})\right]}$$

(b3) Division period efficiency:

(12) $$\rho_{0}^{*}=\min\frac{1-\frac{1}{m_{k}+linkin_{k}+ninput_{k}}(\sum_{i=1}^{m_{k}}\frac{S_{iok}^{t-}}{x_{iok}^{t}}+\sum_{{\left(kh\right)}_{l}=1}^{linkin_{k}}\frac{s_{o{\left(kh\right)}_{l}in}^{t}}{z_{o{\left(kh\right)}_{l}in}^{t}}\sum_{k_{l}}^{ninput_{k}}\frac{s_{ok_{linput}input}^{\left(t,t+1\right)}}{z_{ok_{l}input}^{\left(t,t+1\right)}})}{1+\frac{1}{r_{1k}+r_{2k}}(\sum_{r=1}^{r_{1k}}\frac{s_{rokgood}^{t+}}{y_{rokgood}^{t}}+\;\sum_{r=1}^{r_{2k}}\frac{s_{rokbad}^{t-}}{y_{rokbad}^{t}})}$$

From the above, the overall efficiency, period efficiency, division efficiency, and division period efficiency are obtained for the 30 provinces from 2013–2016.

Our study follows Hu and Wang’s [6] total-factor energy efficiency index to overcome any possible bias in the traditional energy efficiency indicator. There are eleven key features of this present study: labor efficiency, energy consumption efficiency, GDP efficiency, CO_2_ efficiency, PM2.5 efficiency, PM10 efficiency, medical institution assets efficiency, mortality rate efficiency, respiratory disease rate efficiency, technological innovation efficiency (TIE) for energy conservation, and TIE for respiratory medical treatment. In our study, “i” represents area and “t” represents time. The eleven efficiency models are defined in the following:

(13) $$\mathrm{Labor}\;\mathrm{efficiency}\;=\frac{\mathrm{Target}\;\mathrm{Labor}\;\mathrm{input}\;\left(i,t\right)}{\mathrm{Actual}\;\mathrm{Labor}\;\mathrm{input}\;\left(i,t\right)}$$

(14) $$\mathrm{Energy}\;\mathrm{consumption}\;\mathrm{efficiency}\;=\frac{\mathrm{Target}\;\mathrm{Energy}\;\mathrm{consumption}\;\mathrm{input}\;\left(i,t\right)}{\mathrm{Actual}\;\mathrm{Energy}\;\mathrm{consumption}\;\mathrm{input}\;\left(i,t\right)}$$

(15) $$\mathrm{TIE}\;\mathrm{for}\;\mathrm{energy}\;\mathrm{conservation}\;=\frac{\mathrm{Target}\;\mathrm{Energy}\;\mathrm{conservation}\;\mathrm{knowledge}\;\mathrm{input}\;\left(i,t\right)}{\mathrm{Actual}\;\mathrm{Energy}\;\mathrm{conservation}\;\mathrm{knowledge}\;\mathrm{input}\;\left(i,t\right)}$$

(16) $$\mathrm{GDP}\;\mathrm{efficiency}\;=\frac{\mathrm{Actual}\;\mathrm{GDP}\;\mathrm{desirable}\;\mathrm{output}\;\left(i,t\right)}{\mathrm{Target}\;\mathrm{GDP}\;\mathrm{desirable}\;\mathrm{output}\;\left(i,t\right)}$$

(17) $${\mathrm{CO}}_{2}\;\mathrm{efficiency}\;=\frac{{\mathrm{Target}\;\mathrm{CO}}_{2\;}\mathrm{Undesirable}\;\mathrm{output}\;\left(i,t\right)}{{\mathrm{Actual}\;\mathrm{CO}}_{2}\mathrm{Undesirable}\;\mathrm{output}\;\left(i,t\right)}$$

(18) $$\mathrm{PM}2.5\;\mathrm{efficiency}\;=\frac{\mathrm{Target}\;\mathrm{Pm}2.5\;\mathrm{Undesirable}\;\mathrm{output}\;\left(i,t\right)}{\mathrm{Actual}\;\mathrm{Pm}2.5\;\mathrm{Undesirable}\;\mathrm{output}\;\left(i,t\right)}$$

(19) $$\mathrm{PM}10\;\mathrm{efficiency}\;=\frac{\mathrm{Target}\;\mathrm{Pm}10\;\mathrm{Undesirable}\;\mathrm{output}\;\left(i,t\right)}{\mathrm{Actual}\;\mathrm{Pm}10\;\mathrm{Undesirable}\;\mathrm{output}\;\left(i,t\right)}$$

(20) $$\mathrm{Medical}\;\mathrm{institution}\;\mathrm{assets}\;\mathrm{efficiency}\;=\frac{\mathrm{Target}\;\mathrm{Medical}\;\mathrm{institution}\;\mathrm{assets}\;\mathrm{input}\;\left(i,t\right)}{\mathrm{Actual}\;\mathrm{Medical}\;\mathrm{institution}\;\mathrm{assets}\;\mathrm{input}\;\left(i,t\right)}$$

(21) $$\mathrm{TIE}\;\mathrm{for}\;\mathrm{respiratory}\;\mathrm{medical}\;\mathrm{treatment}\;=\frac{\mathrm{Target}\;\mathrm{Respiratory}\;\mathrm{medical}\;\mathrm{treatment}\;\mathrm{knowledge}\;\mathrm{input}\;\left(i,t\right)}{\mathrm{Actual}\;\mathrm{Respiratory}\;\mathrm{medical}\;\mathrm{treatment}\;\mathrm{knowledge}\;\mathrm{input}\;\left(i,t\right)}$$

(22) $$\mathrm{Mortality}\;\mathrm{rate}\;\mathrm{efficiency}\;=\frac{\mathrm{Target}\;\mathrm{Mortality}\;\mathrm{rate}\;\mathrm{output}\;\left(i,t\right)}{\mathrm{Actual}\;\mathrm{Mortality}\;\mathrm{rate}\;\mathrm{output}\;\left(i,t\right)}$$

(23) $$\mathrm{Respiratory}\;\mathrm{disease}\;\mathrm{rate}\;\mathrm{efficiency}\;=\frac{\mathrm{Target}\;\mathrm{Respiratory}\;\mathrm{disease}\;\mathrm{rate}\;\mathrm{output}\;\left(i,t\right)}{\mathrm{Actual}\;\mathrm{Respiratory}\;\mathrm{disease}\;\mathrm{rate}\;\mathrm{output}\;\left(i,t\right)}$$

If the target labor, energy consumption, energy conservation knowledge, medical institution assets, and respiratory medical treatment knowledge inputs equal the actual inputs, then they all equal 1, indicating overall efficiency. If a target input is less than the actual input, then the efficiency is less than 1, indicating overall inefficiency.

If the target undesirable outputs (CO_2_, PM2.5, PM10, mortality rate, and respiratory disease rate) equal the actual undesirable outputs, then their efficiencies all equal 1, indicating overall efficiency. If a target undesirable output is less than the actual undesirable output, then its efficiency is less than 1, indicating overall inefficiency.

If the target GDP desirable output is equal to the actual GDP desirable output, then the GDP efficiency equals 1, indicating overall efficiency. If the actual GDP desirable output is less than the target GDP desirable output, then the GDP efficiency is less than 1, indicating overall inefficiency.

**Figure 1:**
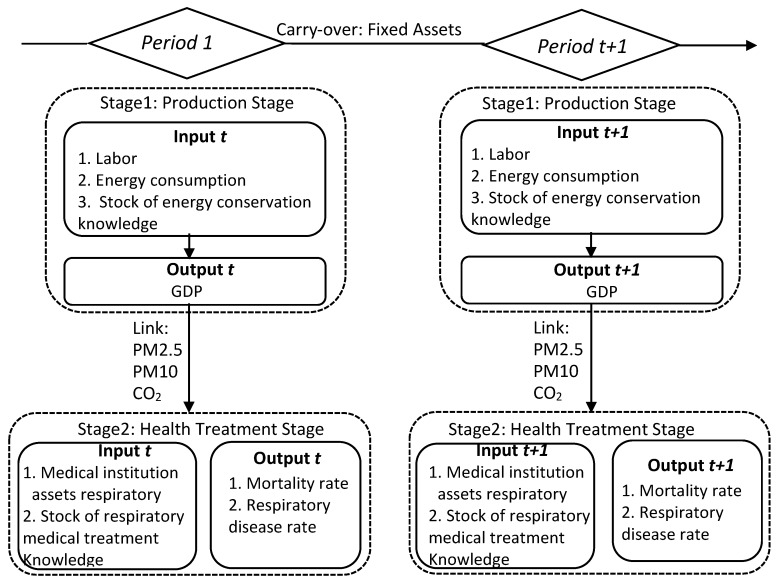
Two-stage undesirable dynamic data envelopment analysis (DEA) model.

**Notes:** GDP is the abbreviation for gross domestic product; PM2.5 is the abbreviation for particulate matter whose diammeter is smaller than 2.5; PM10 is the abbreviation for particulate matter whose diammeter is smaller than 10.

Figure 1 reveals the framework of the modified two-stage dynamic undesirable data envelopment DEA model of inter-temporal efficiency measurement and variables in this study.

## 4. Empirical Study

### 4.1. Data Sources and Description

This study explores the energy and health efficiencies with the inclusion of technological innovation in 30 provinces of China from 2013 to 2016. We employ patent data from the National Intellectual Property Administration to construct the stock of technological innovation. Other data, if not mentioned specially, are collected from the Statistical Yearbook of China. All details are as follows.

In this study, the input and output variables are shown in Table 1. The first stage is production with three input variables and one output variable. The second stage is health treatment with one input variable and two output variables.

#### 4.1.1. First Stage: Production

##### Input Variables

Labor: the number of employees in each province at the end of each year. Unit: 10,000 persons.

Energy consumption: energy consumption in each province each year. Unit: 10,000 tons standard coal equivalent.

Stock of energy conservation knowledge: the knowledge stock of energy conservation calculated from energy conservation patent.

Some scholars use research and development (R&D) investment data to calculate the stock of knowledge by the perpetual inventory method. Two disadvantages in this method should be noted. Firstly, R&D investment stands for the input of technology innovation and not technology itself. Thus, the connection between R&D investment and the knowledge stock is not as close as a patent. Secondly, knowledge stock is different from capital stock. The maximum value of a new invention is not in the current year when the invention is created, because it takes time for the new knowledge to spread widely throughout the economy. The knowledge embodied in a patent also becomes obsolete over time, because new and better inventions are created quickly and frequently. Considering these two issues of decay and diffusion of a patent and following Popp [43,44,45], the stock of knowledge is measured by:

(24) $$K_{t}=\sum_{s=0}^{\infty }e^{-\beta_{1}(s)}\left(1-e^{-\beta_{2}\left(s+1\right)}\right)PAT_{t-s}$$

where $\beta_{1}$ is a rate of decay to capture the obsolescence of old patents, and $\beta_{2}$ is a rate of diffusion to capture the spread of knowledge. The rate of diffusion is multiplied by s+1 to avoid being zero in the current year. According to Popp [43], the decay rate and the diffusion rate are appointed to be 0.1 and 0.25, respectively, implying that the maximum effect of a new patent on the knowledge stock occurs about four years after it is granted, as commonly found in the literature on technological innovation (for example, Aghion [46]; Griliches [47]).

It is a vital issue to define the IPC codes for energy conservation technology. According to Green Inventory published by WIPO in 2010, we define IPC codes for energy conservation technology and collect patent panel data from the National Intellectual Property Administration, PRC (CNIPA) database. We use annual applications of inventions in each province from 2000 to 2016 to construct the stock of knowledge.

##### Output Variables

GDP: GDP in each province each year. Unit: 100 million Chinese yuan(CNY).

#### 4.1.2. Second Stage: Health Treatment 

##### Input Variables

Medical institution assets: capital stock of medical institutions in each province at the end of each year. Unit: 100 million Chinese yuan (CNY).

Stock of respiratory medical treatment knowledge: the knowledge stock of respiratory medical treatment is calculated by using patent data. The calculation method is the same as knowledge stock of energy conservation. A patent of respiratory medical treatment technology is defined as the patents in A61 in the IPC codes with key words asthma, bronchitis, chronic cough, and respiratory disease. The data source is the CNIPA database.

##### Output Variables

Mortality rate: mortality rate in each province each year. Unit: percent.

Respiratory disease rate: number of respiratory patients per 100,000 people. Unit: person.

#### 4.1.3. Variables Linking Production Stage and Health Treatment Stage

CO_2_: carbon dioxide emissions in each province each year. Unit: ton.

PM2.5: the measured PM2.5 in each province each year. Unit: micrograms per cubic meter.

PM10: the measured PM10 in each province each year. Unit: micrograms per cubic meter.

#### 4.1.4. Carryover

Fixed assets: capital stock in each province is calculated by the perpetual inventory method, using fixed assets investment in each province each year. Unit: 100 million RMB.

### 4.2. Input and Output Variables’ Statistical Analysis

**Figure 2:**
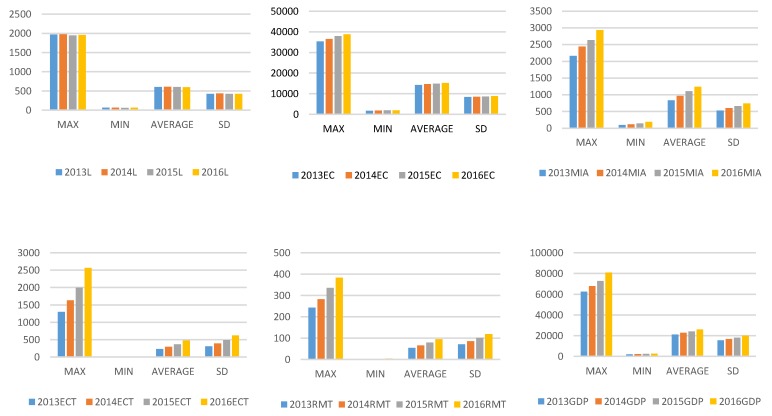

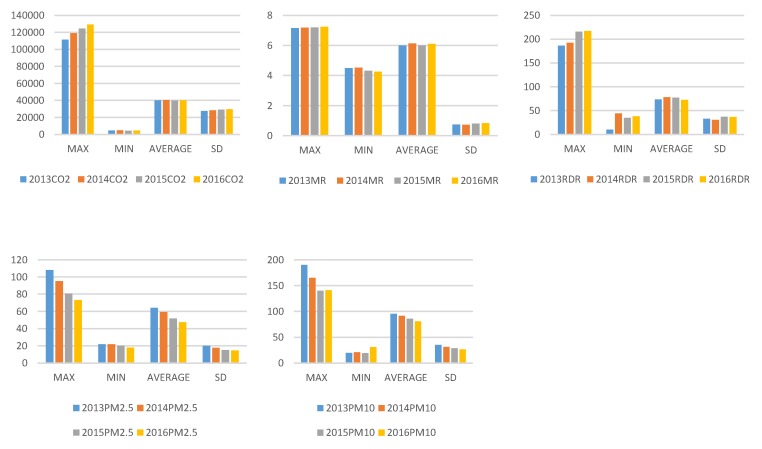
Statistics of input and output variables.

Figure 2 illustrates a statistical picture of the overall variables in stage 1 and stage 2 of the 30 provinces from 2013 to 2016. The average values of labor (L), CO_2_, and mortality rate (MR) do not show an obvious change. The average values of medical institution assets(MIA), knowledge stock of energy conservation(ECT), knowledge stock of respiratory medical treatment(RMT), energy consumption (EC) and GDP increase year after year. The average values of PM2.5 and PM10 decrease year by year. The average values of respiratory disease rate (RDR) move upward first and downward after 2014.

The maximum values of PM2.5 and PM10 decrease significantly. The maximum values of other variables except labor (L) and mortality rate (MR) increase year by year. The minimum values of all variables do not change too much except for respiratory disease rate.

### 4.3. Overall Efficiencies and Ranking with and without Technological Innovation

Table 2 shows the overall efficiencies and ranking of the 30 provinces with and without technologies as input variables. According to economic level and geographic location, we divide the 30 provinces into two parts: eastern region and non-eastern region. The eastern coastal provinces in China have higher economic levels than the non-east region provinces do. The mean of overall efficiencies and ranks with and without technologies in the eastern region are significantly higher than those in the non-eastern region. However, the improvement of efficiency with the inclusion of technological innovations in the east region is lower than that of the non-east region. Relative to excluding technological innovations, the mean overall efficiency score with the inclusion of technological innovations in the eastern region increases slightly from 0.8237 to 0.8898. The mean overall efficiency in the non-eastern region increases from 0.6340 to 0.8037. The mean of the ranking improvement in the non-eastern region also rises higher than that in the eastern region. This indicates the improvements of overall energy and health efficiencies in the economically backward area are higher when including technological innovations.

### 4.4. Annual Overall Efficiencies

Table 3 shows the overall efficiencies in each province from 2013 to 2016. The average efficiencies of all DMUs are increasing slowly. Some DMUs’ efficiencies fluctuate intensely. The average annual efficiencies in the eastern region are higher than those in the non-eastern region in all four years. Those on the efficiency frontier in all four years are Beijing, Fujian, Guangdong, Hainan, Hebei, Inner Mongolia, Ningxia, Qinghai, Shandong, Shanxi, Shanghai, Tianjin, and Yunnan. The annual efficiencies in Guangxi and Chongqing increase from about 0.8 in 2013 to 1 in 2016. The efficiencies in Henan increase from 0.87 in 2013 to 1 after 2015. The overall efficiencies in Guizhou, Jiangxi, and Zhejiang improve in the four years, but still have not reached the frontier yet. This indicates the overall efficiencies in most provinces have improved. However, the overall efficiency in Sichuan decreases from 1 in 2013 to 0.78 in 2016. The overall efficiencies in Liaoning and Heilongjiang are around 0.5 in all four years. This means these two provinces lag behind in overall efficiency and show no signs of improvement. This also coincide with the economic slowdown in these two provinces.

### 4.5. Two-Stage Dynamic Efficiencies

Table 4 shows the energy and health efficiencies in each stage from 2013 to 2016. The results present a rich diversity of performance in all DMUs. The average efficiencies in the production stage (stage 1) are higher than those in the health treatment stage (stage 2). There are 14 provinces whose energy and health efficiencies equal 1 in all four years: Beijing, Fujian, Guangdong, Hainan, Hebei, Inner Mongolia, Ningxia, Qinghai, Shandong, Shanxi, Shanghai, Tianjin, Xinjiang, and Yunnan. Some provinces have efficiencies of 1 in the first stage in all four years, but have efficiency loss in the second stage, such as Guangxi, Henan, Hubei, Hunan, Jiangxi, Zhejiang, and Chongqing. This indicates these provinces should focus on improving health efficiency relative to energy efficiency. The efficiencies in Guangxi, Henan, and Chongqing in the second stage increase quickly and equal 1 in 2016. The efficiencies in Hunan, Jiangxi, and Zhejiang in the second stage increase slowly and do not achieve efficiencies of 1 until 2016. This means the health efficiency in these six provinces has improved. Hubei is the only one whose efficiencies decrease in the second stage, with efficiencies of 1 in the first stage. This indicates Hubei is on the production frontier but is facing public health problems. Therefore, Hubei has a significant need to improve its health efficiency. The energy efficiencies in Heilongjiang and Liaoning are above 0.7 for most years, but their health efficiencies are around 0.3. This indicates that low health efficiency is the root of low overall efficiency in these two provinces.

For all DMUs, both the energy and health efficiencies increase on average during 2013–2016, although some fall back in certain years. The average energy and health efficiencies in the eastern region are both higher than those in the non-eastern region in all four years. The efficiency gap between the eastern and non-eastern regions in the first stage is small. The efficiency gap between the eastern and non-eastern regions in the second stage rises from 0.08 to 0.12 during 2013–2016, because the average efficiency score in the non-eastern region in the second stage has no obvious improvement relative to the eastern region. Therefore, the economically backward area should take more measures on improving health efficiency relative to energy efficiency.

### 4.6. Input Variables’ Efficiencies

Table 5 shows the efficiencies for input variables. They are labor, energy consumption, and medical institution assets. Fourteen DMUs maintain the efficiency frontier for the three input variables from 2013 to 2016: Beijing, Fujian, Guangdong, Hainan, Hebei, Inner Mongolia, Ningxia, Qinghai, Shandong, Shanxi, Shanghai, Tianjin, Xinjiang, and Yunnan. This means almost half the provinces in China are at the efficiency frontier for the three input variables. The efficiencies for labor and energy consumption in Guangxi, Henan, Hubei, Hunan, Jilin, Jiangsu, Jiangxi, Sichuan, and Zhejiang equal 1 in all four years. Gansu, Heilongjiang, and Shaanxi have slowly improved their efficiencies for labor and energy consumption but are still below the average in 2016. These three provinces lag behind and urgently need to improve their efficiencies for labor and energy consumption. Liaoning is the only province whose efficiencies for labor and energy consumption fall quite distinctly.

The efficiencies for medical institution assets in Anhui, Heilongjiang, Hunan, Hubei, Jilin, Jiangsu, and Liaoning fluctuate intensely. The highest efficiencies in these DMUs in the four years is 1, and the lowest score is only 0.22. Five provinces have improved their efficiencies for medical institution assets. Guangxi, Henan, and Chongqing achieve a huge improvement in the four years and finally get an efficiency score of 1 in 2016. Jiangxi and Guizhou have improved a lot in their efficiencies for medical institution assets but have not yet reached the efficiency frontier. Shaanxi and Sichuan are the only two provinces whose efficiencies for medical institution assets show an obvious drop in the four years.

The average efficiencies for labor and energy consumption have maintained a high level, but the average efficiencies for medical institution assets still have broad room to improve, although they have been getting better. The efficiency gaps in labor and energy consumption between the eastern and non-eastern regions are smaller than the efficiency gap in the medical institution assets. The former is decreasing, while the latter is increasing slowly except in 2015. This indicates the economic backward area also lags behind in the efficiencies for the three input variables, especially the efficiency for medical institution assets.

### 4.7. Technological Innovation Efficiencies for Energy Conservation and Respiratory Medical Treatment

The technological innovation efficiencies for energy conservation and respiratory medical treatment have different performances, as shown in Table 6. The technological innovation efficiencies for energy conservation in most DMUs equal 1 in all four years. They include Beijing, Fujian, Guangdong, Guangxi, Hainan, Hunan, Hubei, Henan, Hebei, Jilin, Jiangsu, Jiangxi, Inner Mongolia, Ningxia, Qinghai, Shandong, Shanxi, Shanghai, Sichuan, Tianjin, Xinjiang, Yunnan, and Zhejiang. Guizhou and Chongqing have efficiencies of 1 after 2014. The technological innovation efficiencies for energy conservation in Anhui, Heilongjiang, Liaoning, and Shaanxi decrease a lot, and the lowest efficiency is just 0.3265. There is significant need to promote technological innovation efficiencies for energy conservation in these provinces.

Only fifteen DMUs’ technological innovation efficiencies for respiratory medical treatment equal 1 in all four years. They are Beijing, Fujian, Guangdong, Hainan, Hebei, Inner Mongolia, Ningxia, Qinghai, Shandong, Shanxi, Shanghai, Tianjin, Xinjiang, and Yunnan. Jiangxi, Zhejiang, Guangxi, and Chongqing have improved their technological innovation efficiencies for respiratory medical treatment. Heilongjiang, Liaoning, and Shaanxi’s efficiencies stay at a low level in all four years, and the average efficiencies are just 0.3634, 0.1518 and 0.1997. This indicates these three provinces lag behind in both technological innovation efficiencies.

The average technological innovation efficiencies for respiratory medical treatment are lower than that for energy conservation but show an obvious increasing trend. The average technological innovation efficiencies for energy conservation in the eastern region are similar to those in the non-eastern region without a great gap. The difference in average technological innovation efficiencies for respiratory medical treatment between the eastern and non-eastern regions are larger than that for energy conservation. Therefore, economic backward areas need to catch up with the eastern region in technological innovation, especially for respiratory medical treatment.

### 4.8. Output Variables’ Efficiencies

Table 7 shows the efficiencies for GDP, respiratory disease rate, and mortality rate from 2013 to 2016. Obviously, the efficiency scores of GDP in all DMUs are 1 in all four years, with no efficiency loss. This coincides with the GDP oriented development in China’s provinces. The numbers of DMUs who have efficiency of 1 for respiratory disease rate and mortality rate in all four years are 16 and 14 respectively. This indicates mores provinces have space to improve their efficiency in mortality and respiratory disease rate.

The efficiencies for respiratory disease rate in Anhui, Jiangsu, Zhejiang and Chongqing maintain in frontier except in 2015. This indicates these four provinces should pay attention to the problems of respiratory disease. Guangxi, Jilin, Henan, Hunan, Jiangxi and Liaoning experience some efficiency decrease in 2014 or 2015 but return to frontier in 2016.

The mortality efficiencies in each DMU are almost not changed from 2013 to 2016 except Jiangsu. There are 14 DMUs whose mortality rate efficiencies are 1 in all four years. They are Beijing, Fujian, Guangdong, Hainan, Hebei, Inner Mongolia, Ningxia, Qinghai, Shandong, Shanxi, Shanghai, Tianjin, Xinjiang and Yunnan. These DMUs also have good performance in respiratory disease rate, indicating good condition in public health. Guizhou and Liaoning have the low mortality efficiencies in all four years. Heilongjiang and Sichuan are the only two whose mortality efficiencies decrease dramatically in the four years. There is improvement space for mortality in these four provinces.

The average efficiencies for respiratory disease rate and mortality rate in the eastern region are all higher than those in the non-eastern region in all four years. This indicates the economic backward area also have poor public health. 

## 5. Conclusions and Implications

This study explores energy efficiency and health efficiency with the inclusion of technological innovation in 30 provinces of from 2013 to 2016. We collect patents in the fields of energy conservation and respiratory medical treatment for each province from 2000 to 2016 and construct the stock of energy conservation and respiratory medical treatment knowledge respectively under the consideration of decay and the diffusion of knowledge. Using a two-stage dynamic undesirable DEA model under VRS, we calculate the overall efficiencies. We also calculate annual efficiencies in each stage and the efficiencies for input variables, technological innovations, and output variables from 2013 to 2016. The main results are as follows:

(1) The mean overall efficiencies and ranks in the eastern region are significantly higher than those in the non-eastern region, no matter if technological innovations are included or not. The overall efficiency gap between the eastern and non-eastern regions with the inclusion of technological innovations shows no sign of narrowing. Liaoning and Heilongjiang lag behind in overall efficiency in all four years.

(2) Most DMUs’ efficiencies in the production stage are higher than those in the health treatment stage. The low health efficiency in Liaoning and Heilongjiang is the root of their low overall efficiency.

(3) The average energy and health efficiencies in the eastern region are both higher than those in the non-eastern region in all four years. The efficiency gaps between the eastern and the non-eastern regions in the first stage are smaller than those in the second stage.

(4) The efficiencies of labor and energy consumption in most DMUs equal 1 in all four years. More DMUs experience efficiency loss for medical institution assets. Liaoning is the only province whose efficiencies for labor and energy consumption fall obviously.

(5) The average technological innovation efficiencies for energy conservation are higher than those for respiratory medical treatment. The efficiency gaps in the technological innovation of energy conservation technology between the eastern region and non-eastern region are smaller than that for respiratory medical treatment.

(6) The efficiencies for GDP in all DMUs equal 1 in all four years. The average efficiencies for both respiratory disease rate and mortality rate in the eastern region are all higher than those in the non-eastern region, but the gap is smaller than the efficiencies for input variables.

China’s provincial governments should transform their GDP oriented development to sustainability-oriented development by implementing health in all policies.

(1) Local governments in China, especially those in the economic lagging behind areas, should take measures to improve efficiencies, both in the production and health treatment stages. Measures should be taken to narrow the efficiency gap between the eastern and non-eastern regions.

(2) Relative to energy efficiency, health efficiency has more space to improve. Governments should take more measures to improve health efficiency, such as promoting clean energy consumption, etc.

(3) Most provinces need to improve their efficiencies for medical institution assets, and respiratory disease rate and mortality rate need to be enhanced. The non-eastern region should take more measures in improving energy and health efficiencies to avoid lagging behind further, and it is more urgent to improve health efficiencies.

(4) Governments give priority to technology innovation. Technological innovation can provide a healthier, cleaner living environment efficiently, and its role in the economic backward region is bigger. Therefore, governments should encourage more technological innovation, especially for respiratory medical treatment.

(5) Public policies, such as energy policy, industrial policy, and health policy, should coordinate to improve energy and health efficiencies for sustainability development.

The results show the great gap between the eastern and non-eastern regions. The research framework herein can be modified to evaluate the technology gap ratio. A meta-frontier DEA of the eastern and non-eastern regions in China should be addressed in future research. The inclusion of good outputs (in conjunction with undesirable outputs) in the health treatment stage is an additional future research line.

## Figures and Tables

**Table 1:** Input and output variables.

	Input Variables	Output Variables	Link	Carry-Over
Stage 1	Labor	GDP	CO_2_	Fixed assets
	Energy consumption		PM2.5	
	Stock of energy conservation knowledge		PM10	
Stage 2	Medical institution assets	Mortality rate		
	Stock of respiratory medical treatment knowledge	Respiratory disease rate		

**Table 2:** Overall efficiency scores and ranking with and without innovations.

Region	DMU	Score (w)	Rank (w)	Score (w/o)	Rank (w/o)	Rank Improvement
non-east	Anhui	0.6867	25	0.6391	18	−7
east	Beijing	1	1	1	1	0
east	Fujian	1	1	1	1	0
non-east	Gansu	0.7328	24	0.5433	25	1
east	Guangdong	1	1	1	1	0
east	Guangxi	0.7804	22	0.5561	23	1
non-east	Guizhou	0.8152	21	0.4625	29	8
east	Hainan	1	1	1	1	0
east	Hebei	1	1	0.5440	24	23
non-east	Henan	0.9419	16	0.4506	30	14
non-east	Heilongjiang	0.5504	29	0.4928	28	−1
non-east	Hubei	0.8291	20	0.5278	27	7
non-east	Hunan	0.6419	27	0.6170	19	-8
non-east	Jilin	0.9451	15	0.8077	13	−2
east	Jiangsu	0.8941	18	0.9051	11	−7
non-east	Jiangxi	0.8503	19	0.6461	17	−2
east	Liaoning	0.5080	30	0.5574	21	−9
non-east	Inner Mongolia	1	1	1	1	0
non-east	Ningxia	1	1	1	1	0
non-east	Qinghai	1	1	1	1	0
east	Shandong	1	1	1	1	0
non-east	Shanxi	1	1	0.5566	22	21
non-east	Shaanxi	0.5740	28	0.6162	20	−8
east	Shanghai	1	1	1	1	0
non-east	Sichuan	0.7762	23	0.5358	26	3
east	Tianjin	1	1	1	1	0
non-east	Xinjiang	1	1	0.8260	12	11
non-east	Yunnan	1	1	0.7229	15	14
east	Zhejiang	0.6783	26	0.6585	16	−10
non-east	Chongqing	0.9149	17	0.7749	14	−3
Average of the east		0.9051	8.6667	0.8517	8.5	−0.1667
Average of the non-east		0.8476	15	0.6788	16.6667	2.6667
	MEAN	0.8706		0.7480		

**Table 3:** Overall efficiency score with innovation during 2013–2016.

Region	DMU	2013	2014	2015	2016	MEAN
non-east	Anhui	0.6996	0.6942	0.6937	0.6764	0.6910
east	Beijing	1	1	1	1	1
east	Fujian	1	1	1	1	1
non-east	Gansu	0.6253	0.6822	0.9370	0.7004	0.7362
east	Guangdong	1	1	1	1	1
east	Guangxi	0.6299	0.6934	0.8313	1	0.7886
non-east	Guizhou	0.5833	1	1	0.7586	0.8355
east	Hainan	1	1	1	1	1
east	Hebei	1	1	1	1	1
non-east	Henan	1	0.7903	1	1	0.9354
non-east	Heilongjiang	0.5236	0.5803	0.5959	0.5281	0.5570
non-east	Hubei	0.7722	0.8142	1	0.7489	0.8338
non-east	Hunan	0.6060	0.6561	0.7054	0.6709	0.6596
non-east	Jilin	1	0.7965	1	1	0.9491
east	Jiangsu	1	1	0.6075	1	0.9019
non-east	Jiangxi	0.7071	0.7501	0.9688	1	0.8565
east	Liaoning	0.5460	0.5404	0.5575	0.4521	0.5240
non-east	Inner Mongolia	1	1	1	1	1
non-east	Ningxia	1	1	1	1	1
non-east	Qinghai	1	1	1	1	1
east	Shandong	1	1	1	1	1
non-east	Shanxi	1	1	1	1	1
non-east	Shaanxi	0.5876	0.6255	0.5741	0.5329	0.5800
east	Shanghai	1	1	1	1	1
non-east	Sichuan	1	1	0.6030	0.6187	0.8054
east	Tianjin	1	1	1	1	1
non-east	Xinjiang	1	1	1	1	1
non-east	Yunnan	1	1	1	1	1
east	Zhejiang	0.6469	0.6077	0.6230	0.8497	0.6818
non-east	Chongqing	0.6797	1	1	1	0.9199
Average of the east		0.9019	0.9035	0.8850	0.9418	0.9080
Average of the non-east		0.8214	0.8550	0.8932	0.8464	0.8540
	MEAN	0.8536	0.8744	0.8899	0.8846	0.8756

**Table 4:** Two-stage dynamic DEA efficiency scores.

DMU	2013 Stage1	2013 stage2	2014 Stage1	2014 stage2	2015 Stage1	2015 stage2	2016 Stage1	2016 stage2
Anhui	0.9048	0.4945	0.8814	0.5070	0.8295	0.5579	0.8204	0.5324
Beijing	1	1	1	1	1	1	1	1
Fujian	1	1	1	1	1	1	1	1
Gansu	0.7265	0.5242	0.7093	0.6550	0.8740	1	0.7119	0.6889
Guangdong	1	1	1	1	1	1	1	1
Guangxi	1	0.2598	1	0.3869	1	0.6626	1	1
Guizhou	0.7222	0.4445	1	1	1	1	1	0.5171
Hainan	1	1	1	1	1	1	1	1
Hebei	1	1	1	1	1	1	1	1
Henan	1	1	1	0.5806	1	1	1	1
Heilongjiang	0.7510	0.2962	0.7492	0.4113	0.8109	0.3809	0.7105	0.3456
Hubei	1	0.5444	1	0. 6284	1	1	1	0.4979
Hunan	1	0.2120	1	0.3123	1	0.4109	1	0.3419
Jilin	1	1	1	0.5930	1	1	1	1
Jiangsu	1	1	1	1	1	0.2150	1	1
Jiangxi	1	0.4142	1	0.5002	1	0.9376	1	1
Liaoning	0.7780	0.3140	0.7971	0.2837	0.8390	0.2762	0.6181	0.2861
Inner Mongolia	1	1	1	1	1	1	1	1
Ningxia	1	1	1	1	1	1	1	1
Qinghai	1	1	1	1	1	1	1	1
Shandong	1	1	1	1	1	1	1	1
Shanxi	1	1	1	1	1	1	1	1
Shaanxi	0.7921	0.3832	0.7626	0.4884	0.7787	0.3694	0.7650	0.3008
Shanghai	1	1	1	1	1	1	1	1
Sichuan	1	1	1	1	1	0.2061	1	0.2373
Tianjin	1	1	1	1	1	1	1	1
Xinjiang	1	1	1	1	1	1	1	1
Yunnan	1	1	1	1	1	1	1	1
Zhejiang	1	0.2939	1	0.2154	1	0.2460	1	0.6994
Chongqing	0.9700	0.3894	1	1	1	1	1	1
MEAN	0.9548	0.7523	0.9633	0.7854	0.9711	0.8088	0.9542	0.8149
EAST	0.9815	0.8223	0.9831	0.8238	0.9866	0.7833	0.9682	0.9155
NONEAST	0.9370	0.7057	0.9501	0.7598	0.9607	0.8257	0.9449	0.7479

**Table 5:** Labor (L), energy consumption (EC), and medical institution assets (MIA) efficiency scores.

NO	DMU	2013L	2014L	2015L	2016L	2013EC	2014EC	2015EC	2016EC	2013MIA	2014MIA	2015MIA	2016MIA
1	Anhui	0.9706	0.9089	0.8722	1	0.9481	1	1	1	0.4754	0.3981	0.4357	0.4296
2	Beijing	1	1	1	1	1	1	1	1	1	1	1	1
3	Fujian	1	1	1	1	1	1	1	1	1	1	1	1
4	Gansu	0.7358	0.6651	0.7218	0.6697	0.8204	0.7875	1	0.8293	0.4767	0.6249	1	0.5718
5	Guangdong	1	1	1	1	1	1	1	1	1	1	1	1
6	Guangxi	1	1	1	1	1	1	1	1	0.2378	0.3231	0.5654	1
7	Guizhou	0.7551	1	1	1	0.5197	1	1	1	0.3958	1	1	0.6652
8	Hainan	1	1	1	1	1	1	1	1	1	1	1	1
9	Hebei	1	1	1	1	1	1	1	1	1	1	1	1
10	Henan	1	1	1	1	1	1	1	1	1	0.5454	1	1
11	Heilongjiang	0.7890	0.7413	0.7595	0.8073	0.7491	0.8025	0.8067	0.6795	0.3078	0.3858	0.4085	0.3913
12	Hubei	1	1	1	1	1	1	1	1	0.3678	0.3823	1	0.4822
13	Hunan	1	1	1	1	1	1	1	1	0.2797	0.3021	0.3914	0.4594
14	Jilin	1	1	1	1	1	1	1	1	1	0.6866	1	1
15	Jiangsu	1	1	1	1	1	1	1	1	1	1	0.3236	1
16	Jiangxi	1	1	1	1	1	1	1	1	0.5220	0.5598	0.8752	1
17	Liaoning	0.9693	0.9474	0.9304	0.7322	0.7743	0.8331	0.8349	0.7605	0.4911	0.4742	0.4976	0.5233
18	Inner Mongolia	1	1	1	1	1	1	1	1	1	1	1	1
19	Ningxia	1	1	1	1	1	1	1	1	1	1	1	1
20	Qinghai	1	1	1	1	1	1	1	1	1	1	1	1
21	Shandong	1	1	1	1	1	1	1	1	1	1	1	1
22	Shanxi	1	1	1	1	1	1	1	1	1	1	1	1
23	Shaanxi	0.8259	0.9069	0.7561	0.8638	0.9614	0.8516	1	0.9385	0.5298	0.6358	0.5023	0.3976
24	Shanghai	1	1	1	1	1	1	1	1	1	1	1	1
25	Sichuan	1	1	1	1	1	1	1	1	1	1	0.2907	0.2633
26	Tianjin	1	1	1	1	1	1	1	1	1	1	1	1
27	Xinjiang	1	1	1	1	1	1	1	1	1	1	1	1
28	Yunnan	1	1	1	1	1	1	1	1	1	1	1	1
29	Zhejiang	1	1	1	1	1	1	1	1	0.2943	0.2912	0.3383	0.6350
30	Chongqing	0.9780	1	1	1	0.94757	1	1	1	0.5187	1	1	1
	MEAN	0.9675	0.9723	0.9680	0.9691	0.9573	0.9758	0.9881	0.9736	0.7633	0.7870	0.8210	0.8266
	EAST	0.9974	0.9956	0.9942	0.9777	0.9812	0.9861	0.9862	0.9800	0.8589	0.8604	0.8362	0.9282
	NONEAST	0.9475	0.9568	0.9505	0.9634	0.9414	0.9690	0.9893	0.9693	0.6995	0.7380	0.8108	0.7589

**Table 6:** Technology innovation efficiencies for energy conservation (EC) and respiratory medical (RM).

NO.	DMU	2013 EC	2014 EC	2015 EC	2016 EC	2013 RM	2014 RM	2015 RM	2016 RM
1	Anhui	0.7956	0.7353	0.6164	0.4612	0.5398	0.6578	0.8208	0.6714
2	Beijing	1	1	1	1	1	1	1	1
3	Fujian	1	1	1	1	1	1	1	1
4	Gansu	0.7725	0.6753	0.9003	0.6366	0.6644	0.7990	1	0.8637
5	Guangdong	1	1	1	1	1	1	1	1
6	Guangxi	1	1	1	1	0.3253	0.47968	0.8476	1
7	Guizhou	0.8917	1	1	1	0.6753	1	1	0.6667
8	Hainan	1	1	1	1	1	1	1	1
9	Hebei	1	1	1	1	1	1	1	1
10	Henan	1	1	1	1	1	0.7627	1	1
11	Heilongjiang	0.7149	0.7039	0.8664	0.6447	0.2990	0.5111	0.4109	0.3693
12	Hubei	1	1	1	1	0.7253	0.9460	1	0.5897
13	Hunan	1	1	1	1	0.1745	0.4201	0.5616	0.2757
14	Jilin	1	1	1	1	1	0.6063	1	1
15	Jiangsu	1	1	1	1	1	1	0.1457	1
16	Jiangxi	1	1	1	1	0.3631	0.4973	1	1
17	Liaoning	0.5905	0.6109	0.7516	0.3617	0.1739	0.2181	0.1716	0.1163
18	Inner Mongolia	1	1	1	1	1	1	1	1
19	Ningxia	1	1	1	1	1	1	1	1
20	Qinghai	1	1	1	1	1	1	1	1
21	Shandong	1	1	1	1	1	1	1	1
22	Shanxi	1	1	1	1	1	1	1	1
23	Shaanxi	0.5891	0.5293	0.5800	0.4925	0.2948	0.4057	0.2833	0.2380
24	Shanghai	1	1	1	1	1	1	1	1
25	Sichuan	1	1	1	1	1	1	0.2272	0.2881
26	Tianjin	1	1	1	1	1	1	1	1
27	Xinjiang	1	1	1	1	1	1	1	1
28	Yunnan	1	1	1	1	1	1	1	1
29	Zhejiang	1	1	1	1	0.2935	0.1436	0.1806	0.7643
30	Chongqing	0.9864	1	1	1	0.3199	1	1	1
	Mean	0.9447	0.9418	0.9572	0.9199	0.7616	0.8149	0.8216	0.8281
	EAST	0.9659	0.9676	0.9793	0.9468	0.8192	0.8216	0.7915	0.9067
	NONEAST	0.9306	0.9247	0.9424	0.9019	0.7232	0.8105	0.8417	0.7757

**Table 7:** GDP, respiratory disease rate (RP) and mortality rate (MR) efficiencies.

		2013 GDP	2014 GDP	2015 GDP	2016 GDP	2013 RP	2014 RP	2015 RP	2016 RP	2013 MR	2014 MR	2015 MR	2016 MR
1	Anhui	1	1	1	1	0.9469	0.9173	0.9034	0.9320	1	1	0.8442	1
2	Beijing	1	1	1	1	1	1	1	1	1	1	1	1
3	Fujian	1	1	1	1	1	1	1	1	1	1	1	1
4	Gansu	0.9359	1	1	1	0.8231	0.8261	1	0.9163	1	1	1	1
5	Guangdong	1	1	1	1	1	1	1	1	1	1	1	1
6	Guangxi	1	1	1	1	0.8799	0.9905	1	1	0.9530	0.934838	0.8676	1
7	Guizhou	1	1	1	1	0.8185	1	1	0.8677	0.7717	1	1	0.5565
8	Hainan	1	1	1	1	1	1	1	1	1	1	1	1
9	Hebei	1	1	1	1	1	1	1	1	1	1	1	1
10	Henan	1	1	1	1	1	0.8826	1	1	1	0.864367	1	1
11	Heilongjiang	1	1	1	1	0.9489	0.9326	0.8932	0.8958	1	0.88695	0.9555	0.9034
12	Hubei	1	1	1	1	0.9920	0.8863	1	0.9531	1	1	1	0.8938
13	Hunan	1	1	1	1	0.8578	0.8219	0.8615	0.9299	1	0.865312	0.8192	0.9198
14	Jilin	1	1	1	1	1	0.9188	1	1	1	0.90114	1	1
15	Jiangsu	1	1	1	1	1	1	0.8176	1	1	1	1	1
16	Jiangxi	1	1	1	1	0.8633	0.8867	1	1	1	1	1	1
17	Liaoning	1	1	1	1	0.8819	0.8713	0.8011	0.8344	1	0.688193	0.7758	1
18	Inner Mongolia	1	1	1	1	1	1	1	1	1	1	1	1
19	Ningxia	1	1	1	1	1	1	1	1	1	1	1	1
20	Qinghai	1	1	1	1	1	1	1	1	1	1	1	1
21	Shandong	1	1	1	1	1	1	1	1	1	1	1	1
22	Shanxi	1	1	1	1	1	1	1	1	1	1	1	1
23	Shaanxi	1	1	1	1	0.8480	0.8676	0.8734	0.8868	1	1	1	1
24	Shanghai	1	1	1	1	1	1	1	1	1	1	1	1
25	Sichuan	1	1	1	1	1	1	0.7847	0.8209	1	1	0.7019	0.8553
26	Tianjin	1	1	1	1	1	1	1	1	1	1	1	1
27	Xinjiang	1	1	1	1	1	1	1	1	1	1	1	1
28	Yunnan	1	1	1	1	1	1	1	1	1	1	1	1
29	Zhejiang	1	1	1	1	1	1	1	0.9993	1	0.981276	0.8909	1
30	Chongqing	1	1	1	1	0.8467	1	1	1	1	1	1	1
	Mean	0.9979	1	1	1	0.9569	0.9601	0.9645	0.9679	0.9908	0.9707	0.9618	0.9710
	EAST	1	1	1	1	0.9788	0.9798	0.9682	0.9861	1	0.9725	0.9722	1
	NON EAST	0.9964	1	1	1	0.9423	0.9469	0.9620	0.9557	0.9847	0.9696	0.9549	0.9516
